# A Comprehensive Dataset of Surface Electromyography and Self-Perceived Fatigue Levels for Muscle Fatigue Analysis

**DOI:** 10.3390/s24248081

**Published:** 2024-12-18

**Authors:** Sara M. Cerqueira, Rita Vilas Boas, Joana Figueiredo, Cristina P. Santos

**Affiliations:** 1 Center for MicroElectroMechanical Systems (CMEMS), University of Minho, 4805-017 Guimarães, Portugal; a88316@alunos.uminho.pt (R.V.B.); joana.figueiredo@dei.uminho.pt (J.F.); 2 LABBELS—Associate Laboratory, 4805-017 Guimarães, Portugal

**Keywords:** sEMG, muscle fatigue, perceived fatigue, dynamic movements

## Abstract

Muscle fatigue is a risk factor for injuries in athletes and workers. This brings relevance to the study of this biochemical process to allow for its identification and prevention. This paper presents a novel dataset for muscle fatigue analysis comprising surface electromyography data from upper-limbs and the subject’s self-perceived fatigue level. This dataset contains 13 h and 20 min of data from 13 participants performing a total of 12 upper-limb dynamic movements (8 uni-articular and 4 complex/compound). This dataset may contribute to the testing of new fatigue detection algorithms and analysis of the underlying mechanisms.

## 1. Introduction

Muscle fatigue is a biochemical process that causes changes in the electrical and mechanical characteristics of the muscle [1]. One of the most noticeable mechanical alterations is the decrease in the force generated by the muscle fibers, which reduces muscle power, induces discomfort, and, when the effort is high and recurrent, can even cause pain and trauma [2]. Muscle fatigue can occur during exercise and labor activities, being one of the main risk factors for work-related musculoskeletal disorders due to repetitive efforts [3]. For example, in the Netherlands, fatigue-derived costs are estimated to be EUR 2.1 billion per year, from which EUR 808 million are derived from productivity loss [3]. During sport activities, muscle fatigue can limit performance and can often be associated with injuries [4,5]. This raises the need to study and predict muscle fatigue in a timely manner.

Surface Electromyography (sEMG) is a widely used technique to assess muscular activity and fatigue [6], since it provides a non-invasive method of measuring the electrical activity resulting from the motor unit action potential propagating along muscle fibers. The classical approach to identify muscle fatigue is to analyze the changes in the characteristics of the acquired sEMG signal. It is well-known that muscle fatigue induces a decrease in the muscle fiber conduction velocity and in the signal’s frequency, while its amplitude increases [6]. Thus, some typical muscle fatigue indicators are the Root Mean Square (RMS), Median Frequency (MDF), and Mean Frequency (MNF) [7].

Although there is a significant body of knowledge and research in this field, some challenges remain when identifying muscle fatigue. In particular, ***(i)*** most of these methods are based on a threshold or pattern analysis, which can vary between subjects and muscles, making it difficult to the identify muscle fatigue [8,9]. ***(ii)*** Some methods are time-consuming and fail during real-time performance [10,11]. Alternatively, some researchers have started investigating the use of deep learning to identify muscle fatigue. However, this type of algorithm requires a substantial amount of data, which are often difficult to obtain, as such trials can be time-consuming and demand specific hardware (sEMG sensors) and anatomic knowledge for correct positioning. Additionally, recruiting participants can be difficult, since they need to experience fatigue.

Two public datasets with upper-body muscle fatigue data were identified [12,13]. However, [12] focused on a single isometric movement consisting of a loaded elbow flexion at 90º. Similarly, while [13] included data from nine muscles, as well as a sports physiotherapist’s manual palpation-based muscle tightness scores to complement the participants’ self-reported perceived fatigue levels, it also only provided data from isometric movements. Therefore, both datasets lack dynamic movements, which reduces their applicability to real-world applications, since everyday movements are mostly dynamic.

In order to address some of these limitations, we present a novel dataset for muscle fatigue classification. It differs from existing literature by providing both dynamic uni-articular movements and task-oriented movements such as shoulder flexion, extension, abduction, elbow flexion, and a compound movement simulating welding; an industrial task. These movements recruit commonly studied upper limb muscles, such as the biceps brachii, anterior deltoid, posterior deltoid, and medius deltoid, from both the right and left arms. The participants performed the movements until they were fatigued. The data were labeled according to each participant’s perception of their fatigue, on a 3-level scale.

This article is divided into three sections: ***(i)*** the methodology used to acquire and process the data; ***(ii)*** the database created with the data and its structure; ***(iii)*** brief validation of the data.

## 2. Methods

This section lays out the collection and processing of the dataset.

### 2.1. Participants

Healthy participants from the University of Minho academic community were contacted to participate in this study. They were informed about the study’s goal, details, protocol, and duration. The inclusion criteria to select the participants were as follows: *(i)* over 18 years of age; *(ii)* full range of motion; *(iii)* no clinical history or evidence of motor injuries, including shoulder or arm pain; and *(iv)* no upper-limb exercises performed within the previous 48 h. Thirteen participants (five females and eight males; age: 23.92 ± 3.36 years old, height: 173 ± 10 cm, body mass: 66.1 ± 10.52 kg), all right-handed, voluntarily agreed to participate in this study. The anthropometrics, as well as the exercise habits and caffeine intake of each participant, can be consulted in Table A1. All of the participants provided their written and informed consent to participate in this study according to the ethical conduct defined by the University of Minho Ethics Committee in Life and Health Sciences (CEICVS 006/2020), following the standards set by the declaration of Helsinki and the Oviedo Convention. The participants’ rights were preserved; therefore, personal information that could be used to identify them remained confidential and it is not provided in this dataset.

### 2.2. Instrumentation and Data Collection

The participants were instructed to not perform any high-intensity physical activity using their upper limbs in the 48 h prior to data acquisition, as they would be excluded from the experiment. To avoid upper limb movement constraints and ease the instrumentation, each participant was instructed to wear loose clothing on their upper body (shirts or strap tops which allowed easy access to upper arm muscles) and sneakers. The data were collected at the School of Engineering of the University of Minho.

**Figure 1:**
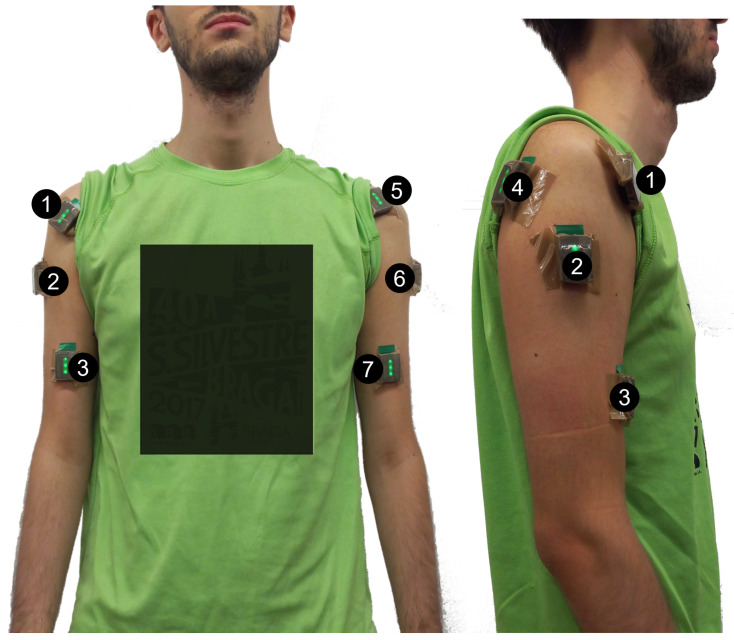
Participant prepared with 8 wireless sEMG sensors (Trigno Avanti, Delsys) placed on: (1) right deltoid anterior, (2) right deltoid medius, (3) right biceps branchii, (4) right deltoid posterior, (5) left deltoid anterior, (6) left deltoid medius, (7) left biceps branchii. Sensor 8 is placed on the left deltoid anterior.

The participants were prepared as follows (Figure 1): *(i)* The skin was shaved and cleaned with a alcohol pad to remove dead skin and natural oils and decrease contact impedance; *(ii)* 8 wireless sEMG sensors (Delsys Trigno Avanti, Delsys Incorporations, USA) were placed on the biceps branchii (BB), deltoid anterior (DA), deltoid posterior (DP), and deltoid medius (DM) of both upper limbs using a double-sided adhesive. The sensors were placed over the muscle belly, with the arrow parallel to the muscle fiber orientation, following SENIAM and the manufacturer’s recomendations [14,15]. To ensure a secure hold and minimize the risk of motion or displacement artifacts, all sensors were taped with *Tesa Pack* tape.

**Figure 2:**
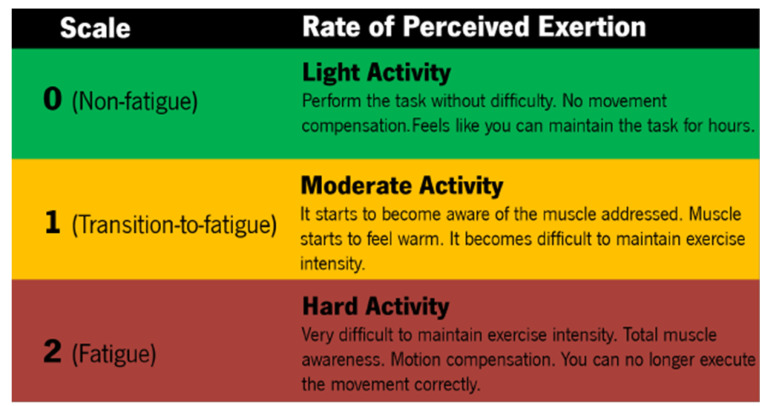
Self-perceived fatigue level description.

All of the participants were prepared by the same researcher to ensure repeatability and minimize errors caused by misplacement. Data collection included: *(i)* electrophysiological signals from the 8 muscles, provided by EMGworks Acquisition from Delsys, at 1259 Hz; *(ii)* participants’ self perceived fatigue level, rated on a 3-level scale (presented in Figure 2) and collected at 50 Hz.

The self-perceived fatigue level was recorded using a custom system developed by our team, consisting of an Arduino Mega with two push buttons. The system started at level 0 (no fatigue) and was updated to level 1 (transition to fatigue) or level 2 (fatigue) when the researcher pressed the corresponding button. The definition of the fatigue scale, shown in Figure 2, was based on the modified Borg scale, which has 10 levels. However, since self-perceived fatigue had to be collected in real time and the participants needed to quickly and confidently assess their fatigue, a 10-level scale was considered to be too demanding. Further, since self-perceived fatigue assessment is subjective in nature and, in some cases, influenced by the participant’s exercise/exertion background, using 10 levels could increase data uncertainty. In addition, a 10-level scale could increase the cognitive load involved in classifying fatigue, potentially leading to delayed responses if the participant becomes uncertain about their fatigue level. These factors could limit the reliability of the data and hinder future applications that require accurate labeling.

This 3-level scale was explained to all the participants by the same researcher to ensure consistent interpretation of muscle fatigue. Throughout the experiment, this researcher regularly asked the participants for feedback on their fatigue state, pressing one of the buttons when the participant reported a different level of fatigue. All of the data were synchronized using a Delsys’s hardware trigger.

### 2.3. Experimental Protocol

First, 3 maximum voluntary contractions of all 8 muscles were acquired following the SENIAM guidelines [14]. The participants were instructed to perform 12 movements in total, consisting of 6 movements per upper limb (4 uni-articular and 2 complex/compound movements), as illustrated in Figure 4. The compound movement was included to provide data that resemble an industrial task, such as welding. This movement selection was intentionally designed to focus on muscle fatigue, as studying simpler, unilateral tasks can provide clearer information on the progression of fatigue.

The participants were informed that the movements should be performed to the point of exhaustion, which means that after reaching fatigue level 2, they were instructed to continue performing the movement until they could no longer endure it. Only one repetition of each movement was recorded, as it was anticipated that subsequent repetitions would be affected by fatigue.

The protocol began with uni-articular movements, selected to predominantly activate the target muscles. The participants performed the movements while holding a 1.5 kg dumbbell to accelerate the onset of fatigue. A metronome set at 30 bpm was used to regulate movement speed. The concept of body–mind connection was explained to the participants, and they were encouraged to focus on it during the exercises. The participants then continued with the compound movements. A weight of 2 kg was placed on each participant’s arm. This weight served the same purpose as the dumbbell but allowed the participants to keep their hands free for movement. The trial involved drawing a pentagon in two arm reaches over a table, one to engage the deltoid anterior and another to engage the deltoid posterior, simulating the continuous motion of welding five lines.

**Figure 3:**
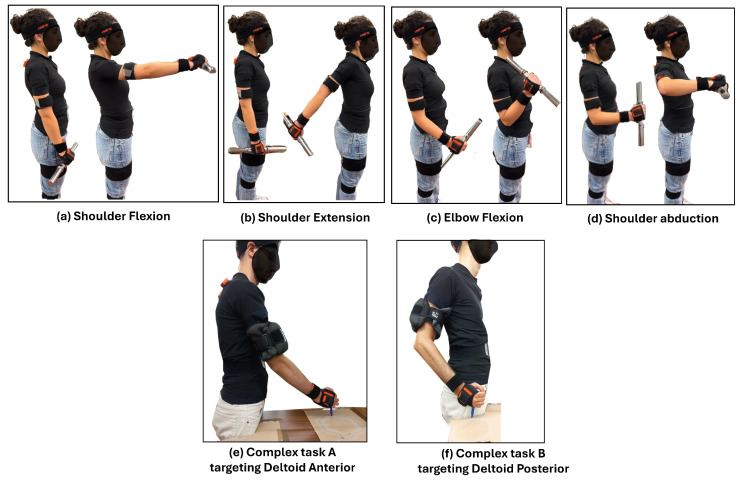
Experimental protocol. All movements were executed, one at a time, with both the right and left arm.

**Figure 4:**
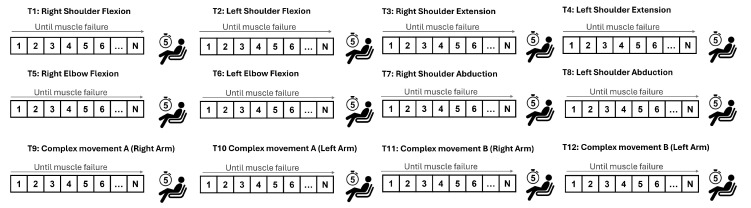
Flowchart illustrating the experimental protocol sequence.

Figure 3 presents the executed movements, namely the four uni-articular and two compound motions. Twelve trials (six for each side) were recorded, and the sequence of movements performed by the participants is illustrated in Figure 4. Signals were collected from both arms, alternately, in order to rest the arm that had previously been under strain. Between trials, a 5 min rest was implemented.

## 3. Results

### 3.1. Data Records

**Figure 5:**
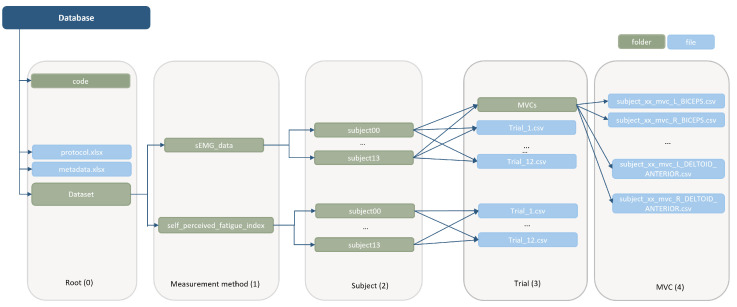
Hierarchical folder structure of the database.

All of the collected data resulting from the protocol described in this paper are available for download on Zenodo [16], allowing reuse across the community. Figure 5 presents the hierarchical organization of the database, which is structured hierarchically in 5 levels. Level 0, the root, hosts the participants’ metadata and acquisition protocol, a folder containing the code used in this manuscript to analyze data, and a second folder containing the dataset. Level 1 contains two folders, each with data corresponding to each measurement method, i.e., sEMG and self-perceived fatigue level data. Level 2 organizes the data into folders for each subject. Level 3 contains the files with the data from the four active muscles during each one of the executed movements (each trial corresponds to one movement) and the folder with the MVCs of the participants. Level 4 contains each participants’ MVC files; one for each muscle. The sEMG data contained in this database correspond to the raw unfiltered sEMG signals obtained from the sensory system.

#### 3.1.1. sEMG Data

**Figure 6:**
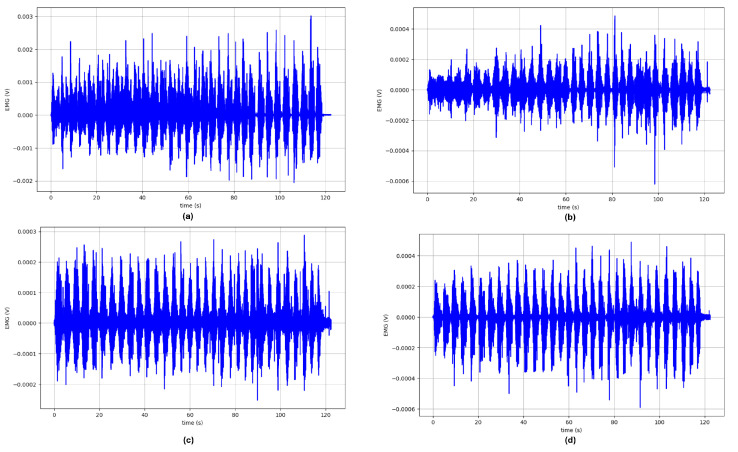
Filtered sEMG signals from participant S02 during trial 2, left shoulder flexion, of the following muscles: (**a**) left biceps branchii, (**b**) right deltoid anterior, (**c**) right deltoid posterior, (**d**) right deltoid medius.

An total of 800.01 min, equivalent to 13 h and 20 min of sEMG data, were collected using the proposed protocol at 1259 Hz. Appendix A, Table A2 presents the time distribution of the data across subjects. All of the data, including the MVC data, were exported to a.csv file using the proprietary Delsys EMGworks Analysis software. These data were then filtered using a 4th-order Butterwoth bandpass filter (20–450 Hz) to remove possible external disturbances such as motion artifacts and/or electrical noise [17]. Since the movements in the protocol involved only one arm, the research team decided to exclude the columns that correspond to the muscles of the inactive arm. That means that only four signals are presented per file/trial. Figure 6 presents the filtered signals from participant S02 during trial 2, corresponding to the flexion of the left shoulder.

#### 3.1.2. Participant’s Self Perceived Fatigue Level

The self-perceived fatigue levels of each participant for each trial are stored as a .csv file. These data were sampled at 50 Hz. Each file contains the elapsed time in seconds since the start of data acquisition and the participant’s self perceived fatigue level, between zero and two. It is noteworthy that while the acquisition start was synchronized across all systems using a hardware device, the stop time was not synchronized. As a result, these files are longer in duration compared to the sEMG.

**Figure 7:**
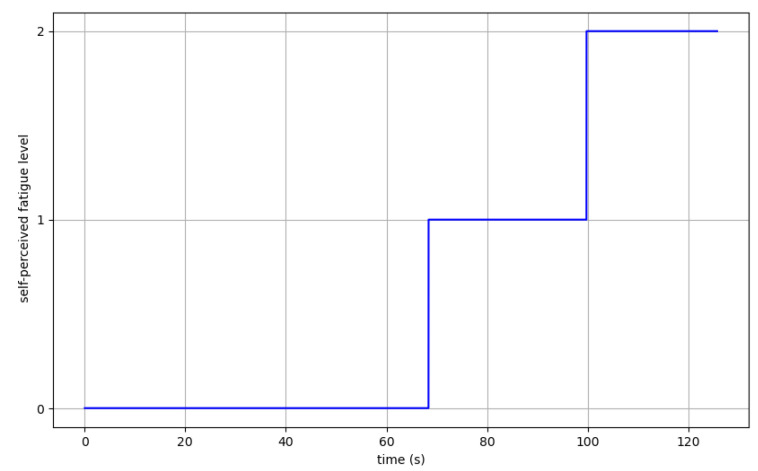
Self-perceived fatigue level evolution from participant S02 during trial 2.

Figure 7 presents an example of the variation in time of the self-perceived fatigue level of participant S02 during trial 2, corresponding to the left shoulder flexion.

## 4. Discussion

### 4.1. Technical Validation

The participants were instructed to follow the outlined protocol. While one researcher supervised and guided the participants’ performance, another researcher visually monitored the data during acquisition. Trials with issues such as sensor failures or data desynchronization were repeated. The data were also reviewed offline.

#### Fatigue Indicator Analysis

To confirm the presence of muscle fatigue in the collected data, a frequency analysis was conducted using two of the most commonly use metrics: the MDF and MNF [18]. These metrics were computed using windows of 4 s, which approximately correspond to one contraction–relaxation cycle (given the 30 bpms cadence), with a 50% signal overlap. Equations (1) and (2) present the definition of MNF and MDF, respectively.

(1) $$\mathrm{MNF}=\frac{\sum_{i=1}^{N}f_{i}P\left(f_{i}\right)}{\sum_{i=1}^{N}P\left(f_{i}\right)}$$

(2) $$\mathrm{MDF}=\frac{1}{2}\sum_{i=1}^{N}P\left(f_{i}\right)$$

where $f_{i}$ is the frequency value of the EMG power spectrum at the frequency bin *i*, $P_{i}$ is the EMG power spectrum at the frequency bin *i*, and *N* is the length of the frequency bin.

**Figure 8:**
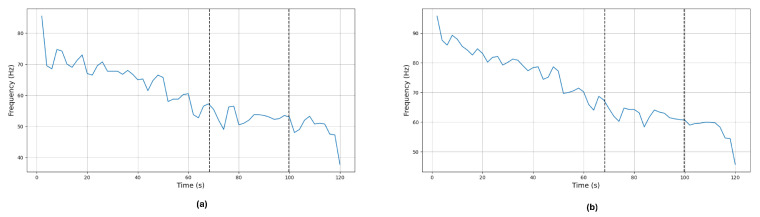
(**a**) MNF signal (**b**) MDF signal of the right anterior deltoid muscle for participant S02 during trial 2 (left shoulder flexion). The black lines represent the changes in self-perceived fatigue level, meaning that level 0 (no fatigue) was perceived until 68 s, level 1 was perceived from 68 s to 99.7 s, and level 2 (fatigue) was perceived from 99.7 s to the end of the trial.

Due to space constraints, only the analysis of the prime-mover muscles in each trial will be depicted. Figure 8 presents the MNF and MDF resulting from participant S02 during trial 2 of the prime-mover muscle; in this case, the right deltoid anterior. A decreasing trend in both MNF and MDF metrics can be observed, as expected in signals where fatigue is present [19].

**Figure 9:**
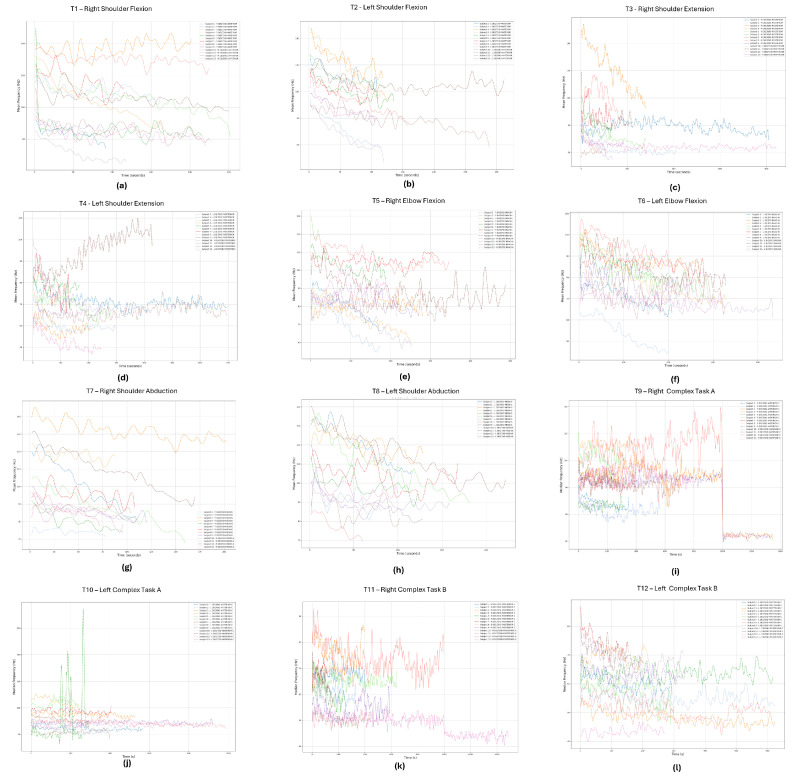
MNF signal evolution of the primer mover of each trial: (**a**) right deltoid anterior, (**b**) left deltoid anterior, (**c**) right deltoid posterior, (**d**) left deltoid posterior, (**e**) right biceps branchii, (**f**) left biceps branchii, (**g**) right deltoid medius, (**h**) left deltoid medius, (**i**) right deltoid anterior, (**j**) left deltoid anterior, (**k**) right deltoid posterior, (**l**) left deltoid posterior. Each line in the figures represents a different participant.

**Figure 10:**
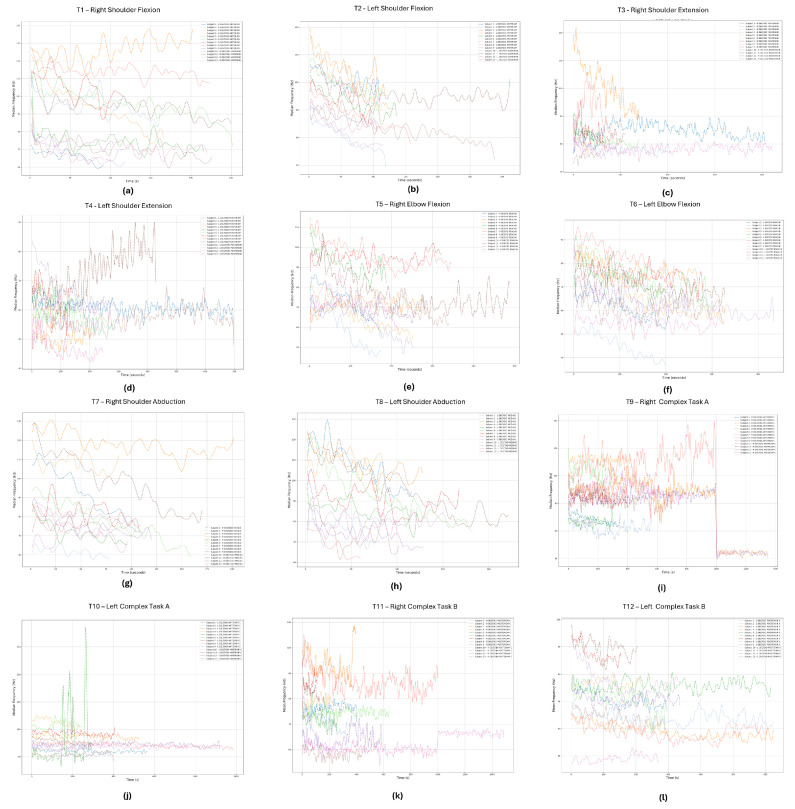
MDF signal evolution of the primer mover of each trial: (**a**) right deltoid anterior, (**b**) left deltoid anterior, (**c**) right deltoid posterior, (**d**) left deltoid posterior, (**e**) right biceps branchii, (**f**) left biceps branchii, (**g**) right deltoid medius, (**h**) left deltoid medius, (**i**) right deltoid anterior, (**j**) left deltoid anterior, (**k**) right deltoid posterior, (**l**) left deltoid posterior. Each line in the figures represents a different participant.

Figure 9 and Figure 10 presents the time evolution of MDF and MNF, respectively. To improve the visualization of these metrics across all subjects and trials, since these dynamic movements result in rapid changes in frequency, a Savitzky–Golay filter was implemented to smooth the data and allow for better visualization of the metrics’ trends. This technique was selected because it smooths the signal without significantly distorting the local patterns or behaviors of the signal’s data points [20].

As expected, the MNF values are slightly higher that the MDF. This occurs due to the skewed shape of the EMG power spectrum; consequently, the variance of MNF is typically lower than that of MDF [21]. The majority of the data depict a decreasing trend in both MDF (Figure 10) and MNF (Figure 9) metrics. The most notorious exceptions are as follows. For participant 11, during t4, the trend of both frequency-based metrics during the experiment goes against the expected behavior, possibly caused by poor body–mind coordination or a slouched posture affecting the movement. Participants 8 and 9, during t9, present an oscillatory frequency behavior with a stable mean. However, near the 1000 s time mark, the frequency drops very quickly. This can also be a sign of movement compensation due to fatigue or even some level of distraction by the participant, due to the long duration of the trial. Lastly, during t10, participant 5 exhibited high-frequency peaks, likely caused by noise or motion artifacts.

Minor exceptions include participant 3 during t1, participants 7 and 11 during t5, participant 12 during t4, and participant 9 during t6. While these participants initially showed a decreasing trend, their frequency values increased mid-trial, which may indicate compensatory movements due to fatigue. Depending on the application, our recommendation is to not use or to be careful using the data from these specific trials, specially the ones with the most notorious exceptions.

### 4.2. Code Availability

This database is accompanied by a Jupyter notebook script that was used to process the data and compute the metrics presented in this paper.

## Data Availability

The data and the code used in this manuscript are available for download at Zenodo.

## Abbreviations

The following abbreviations are used in this manuscript:

sEMG	Surface Electromyography
RMS	Root Mean Square
MDF	Median Frequency
MNF	Mean Frequency
MPF	Mean Power Frequency
BB	Biceps Branchii
DA	Deltoid Anterior
DP	Deltoid Anterior
DM	Deltoid Medius

## Appendix A

**Table A1:** Participants’ anthropometrics, exercise habits, and caffeine intake on the day of the trial.

Participant	Gender	Age (years)	Height (cm)	Weight (kg)	Workouts per Week	Had Coffee Today?
S01	F	27	170	63.5	3	Yes
S02	F	23	153.5	46.8	2	Yes
S03	M	22	184	83	4	Yes
S04	F	23	167	57	6	Yes
S05	M	33	174	68	2	Yes
S06	M	24	177	77	5	Yes
S07	M	20	188	84	2	No
S08	M	27	174	58	2	Yes
S09	F	23	162	65	4	Yes
S010	F	25	163	56	0	Yes
S011	M	22	165	60	6	No
S012	M	20	181	72	0	No
S013	M	22	188	69	0	No

**Table A2:** Time distribution of data across subjects.

Participant	Total Time (min)
S01	66.14
S02	57.65
S03	77.10
S04	39.13
S05	46.14
S06	52.08
S07	41.02
S08	84.29
S09	77.03
S010	43.69
S011	56.14
S012	67.83
S013	91.65
**Total**	**800.01**
